# Risk assessment based on spectrophotometric signals used in eco-friendly analytical scenarios for estimation of carvedilol and hydrochlorothiazide in pharmaceutical formulation

**DOI:** 10.1038/s41598-024-69746-0

**Published:** 2024-08-23

**Authors:** Mona Nabil, Hoda M. Marzouk, Dina A. Ahmed, Samah S. Abbas, Hayam M. Lotfy

**Affiliations:** 1 Pharmaceutical Chemistry Department, Faculty of Pharmacy, Future University in Egypt, Cairo, 11835 Egypt; 2 Pharmaceutical Analytical Chemistry Department, Faculty of Pharmacy, Cairo University, El-Kasr El-Aini Street, Cairo, 11562 Egypt

**Keywords:** Absorbance resolution (AR), Carvedilol, Greenness profile, Hydrochlorothiazide, Induced absorbance resolution (IAR), Spectrum subtraction (SS), Analytical chemistry, Green chemistry

## Abstract

Special attention is given to the pharmacological treatment of combined medication of Carvedilol and hydrochlorothiazide which is the most effective and the most beneficial therapy for hypertensive patients with diabetes and various metabolic comorbidities. This work represents spectrophotometric platform scenarios based on factorized spectrum (FS) using interpoint data difference resolution scenarios (IDDRS) coupled with spectrum subtraction method (SS) for the concurrent quantification of carvedilol (CAR) and hydrochlorothiazide (HCT) when present together in a combination without the need for any initial physical separation steps. This IDD resolution scenario based on manipulating the zero-order spectra (D^0^) of both drugs in the mixture with various spectral features at different wavelength regions (200–400 nm), region I (220–250 nm), region II (240–300 nm) and region III (270–320 nm) via absorbance resolution (AR) and induced absorbance resolution (IAR) methods coupled with corresponding spectrum subtraction (SS). The calibration curves were established across the linearity ranges of 2.0–12.0 µg/mL at 242.50 nm and 4.0–40.0 µg/mL at 285.5 nm for CAR and 1.0–11.0 µg/mL at 226.10 nm and 2.0–20.0 µg/mL at 270.5 nm for HCT. Moreover, methods’ validation was confirmed via ICH guidelines. A Multicenter comparison between sensitivity, specificity in respect resolution sequence were applied using different wavelength regions with various concentration ranges was applied and finally spectral resolution recommendation is issued and cumulative validation score (CVS) is calculated as an indicator in the risk analysis. In quality control laboratories, the studied approaches are applicable for conducting analysis on the mentioned drugs. In addition, the selection of spectrophotometry aligns with the principles of green analytical chemistry, an approach that resonates with the overarching theme of minimizing environmental impact. Via four metric tools named: analytical greenness (AGREE), green analytical procedure index (GAPI), analytical eco-scale, and national environmental method index (NEMI), methods’ greenness profile was guaranteed.

## Introduction

Hypertension poses a significant worldwide health concern, impacting millions of individuals for being the foremost avoidable risk factor for atherosclerosis and ischemic heart disease. Nowadays, hypertensive patients may require a combination of antihypertensive medications instead of utilizing a single antihypertensive treatment to ensure a significant decrease in blood pressure records, improved target organs’ protection with a concurrent decrease in adverse effects^1^.

Hypertension and type 2 diabetes commonly coexist as comorbidities. The incidence of hypertension is two times higher in individuals who have diabetes in comparison to those without diabetes. Additionally, individuals diagnosed with hypertension frequently show manifestations of insulin resistance, rendering them more prone to diabetes in comparison to those who have normal blood pressure. Furthermore, there is a significant intersection in the cardiovascular complications of hypertension and diabetes, predominantly associated with macrovascular and microvascular disorders^2^.

*Carvedilol (CAR)*, 1-(9H-carbazol-4-yloxy)-3-[[2-(2-methoxyphenoxy) ethyl] amino]-2-propanol^3^; Fig. S1a. CAR, classified as a potent antihypertensive agent, belonging to third-generation beta-blockers and characterized with alpha1-blocking action. Notably, it exhibits more advantageous effects, particularly in difficult metabolic conditions. Unlike traditional beta-blockers, CAR decreases blood pressure (BP) by attenuating vascular resistance while maintaining cardiac output and has a diminished impact on heart rate. Moreover, alternative beta-blockers such as metoprolol or atenolol, resulted in considerable weight gain in contrast to CAR^4^. According to many studies, CAR was found to be more effective than metoprolol or atenolol in enhancing lipid and glucose metabolic parameters and attenuating lipid peroxidation in hypertensive diabetic patients. Owing to its alpha-receptor-blocking properties, carvedilol has a significant vasodilatory impact, potentially explaining its antidiabetic effect^5–8^.

Literature survey revealed that CAR’s determination either alone or when combined with other drugs was applied through titrimetric^9,10^, spectrophotometric^11^ and chromatographic^12^ methods.

*Hydrochlorothiazide* (*HCT*), 6-chloro-3,4-di hydro-2H-1,2,4-benzothiadiazine-7-sulfonamide1,1-dioxide^3^; Fig. S1b. HCT belongs to the thiazide class of diuretics. It inhibits the electroneutral Na^+^–Cl^–^ cotransporter located on the apical membrane of the early segment of the distal convoluted tubule^13^. HCT’s determination, either alone or when combined with other drugs was accomplished utilizing titrimetric^14^, spectrophotometric^15^ and chromatographic^9,16^ methods.

Co-Dilatrol^®^ Tablet; is cardiovascular medicine containing CAR and HCT. Taken together, a marked synergistic pharmacological effect is achieved for managing the elevated blood pressure in a shortest possible time with maximum patient’s satisfaction^17^. Hence, several studies have demonstrated the metabolic neutrality or potential metabolic benefits subsequent to carvedilol therapy in patients with diabetic hypertension^18–20^.

Reviewing the literature demonstrated several methods for analyzing CAR and HCT simultaneously in their co-formulated pharmaceutical preparations including spectrophotometric^3,21–24^, HPLC^25–29^, HPTLC^30^ and CE^31^ methods.

Analytical methodologies relying on measurements of UV absorption are widely recognized as among the most popular and frequently applied methods in laboratory practices. Spectrophotometric techniques are generally not demanding in terms of labor and time. The economic and environmentally friendly features of UV procedures are worthy of emphasis as well.

Novel software-controlled spectrophotometers enable acquisition and storage of recorded spectra, and facilitate in-silico mathematical operations using the different windows of the spectrophotometer platform (zero (I), derivative (II), ratio (III) and manipulated ratio (IV)). UV spectrophotometric methodologies are commonly applied for analysis of multiple components^32–42^. The manipulated spectrum obtained in various windows exhibits a close correlation with the geometric aspects of the scanned zero-order spectrum; hence, it is evident that the process of spectrum registration plays a pivotal approach. Among the various windows, window I concerning manipulation on the zero order spectrum of the mixture exhibits superiority, characterized with minimal limitations and requirements^43,44^.

Recently, the introduction of a resolution tool, namely factorized spectra (FS) into the response data has proven to be a crucial methodology within spectrophotometry^45–47^.The exquisite integration between the manipulations of response data on the spectra along with FS allow the recovery of D^0^ spectra of the studied drugs which acts as fingerprint of the analytes and enable their precise and accurate quantification at their maxima with minimal error. This approach results in a significant improvement of selectivity, comparable to chromatographic techniques, while requiring minimal effort and reduced solvent consumption^45–50^. In addition, the concept of green chemistry is widely recognized in chemical laboratories. To properly measure an environmental impact of analytical method, dedicated assessment tools are required such as an analytical greenness metric tool (AGREE)^49–52^,green analytical procedure index (GAPI)^52–54^, analytical eco-scale^55^ and national environmental method index (NEMI)^56^.

The main goal of our research was focused on revealing how is factorized spectrum prepared via built-in spectrophotometer software used at spectral resolution of drugs in pharmaceutical formulation and which benefits and obstacles are perceived by its usage. Diverse resolution scenarios of spectrophotometric platform applied on zero order spectra (window I) at different wavelength regions (200–400 nm) utilizing factorized spectrum were developed and applied in the analysis of pharmaceutical preparation containing CAR and HCT. The investigated methods underwent validation in accordance with the guidelines outlined by the International Conference on Harmonization (ICH)^57^, demonstrating compliance with the specified acceptance criteria. Analysis of risk of each method at their specific wavelengths is applied which affect the accuracy and precision of the results. The novel score value namely cumulative validation score (CVS) is calculated and used in the assessment of the error risk at various wavelength regions in order to recommend the optimum experimental scenario with optimum accuracy and precision. Ultimately, the studied methods’ implications on the environment and human health were assessed using analytical greenness metric tool AGREE)^49–52^, GAPI^52–54^, analytical eco-scale^55^, and NEMI^56^.

## Background

### Interpoint data difference resolution scenarios (IDDRS) using window I of spectrophotometric platform

This resolution method relies on processing the data from the absorption spectrum of the mixture to achieve both separation and identification through factorized spectrum using recorded or modified absorbance data of window I within linearity ranges of the drugs at the selected wavelengths. The spectra of the investigated drugs are scanned, considering their distinctive features, shape, and the extent of overlapping, whether partial or complete and the presence of any spectrum extensions. The selection of wavelength region should be carried out by considering the geometrical configuration of the target spectrum and spectral characteristics of accompanying compounds. When developing a spectrophotometric method, it is crucial to optimize the parameters of the employed spectrophotometer for reliable and accurate results. Interpoint data difference (IDD) scenario based on the absorbance difference (ΔA)^47^ which can be applied on the recorded absorbance data in absorbance resolution method^47,49,58–60^ or on modified absorbance data (IΔA)^60^ in induced absorbance resolution method (IAR).The parent spectra targeting drug are obtained and the concentrations of X and Y are computed by applying their respective regression equations at their associated absorbance maxima.

#### Absorbance resolution coupled with spectrum subtraction method (AR-SS)

The application of this smart resolution technique via absorbance difference is suitable for analyzing a binary mixture containing components X and Y, characterized by fully overlapped zero-order absorption spectra. In this context, the absorbance difference (ΔA, A_1_–A_2_) between two designated wavelengths on the mixture's zero-order spectra exhibits direct proportionality to the concentration of component X, whereas, for component Y, the absorbance difference fundamentally remains zero. The factorized spectrum of component X (FS_∆A_) of X, expressed as $\frac{{X{ }\left( {D^\circ } \right){ }}}{\Delta A}$, is generated utilizing computational software through the division of the (D^0^) associated with any concentration of pure X within its linearity by the absorbance difference measured at two specified wavelengths where the contribution of the other component is negligible. The sensitivity of the methods based on the absorbance difference values on the spectrum at the selected wavelengths points (interpoint distance ∆ℷ).

Absorbance values are typically measured at two designated wavelengths in laboratory prepared mixtures. The computed absorbance difference (∆A) is subsequently multiplied by the respective factorized spectrum of the target pure drug.

1 $$\Delta A \cdot \frac{{X{ }\left( {D^\circ } \right){ }}}{\Delta A} = {\text{ Recovered }}\left( {{\text{D}}^\circ } \right)o{\text{f }}X$$

The resolution of component Y is accomplished by employing the spectrum subtraction methodology, wherein the derived spectrum (D^0^) of X is subtracted from the spectrum of the combined mixture, X + Y.

#### Induced absorbance resolution coupled with spectrum subtraction method (IAR-SS)

This innovative approach is applicable to the analysis of a binary mixture comprising X and Y, characterized by fully overlapped zero-order absorption spectra at two specified wavelengths, λ_1_ and λ_2_.Importantly, at these wavelengths, the absorbance of the interfering analyte is unequal, indicating a difference in absorbance that is not zero. The equality factor of component Y at the two designated wavelengths (F_Y_) is mathematical factor computed to nullify the influence of Y at these particular wavelengths. Subsequently, the modified absorbance difference between the two specific wavelengths in the spectra of the laboratory mixtures exhibits a direct proportionality to the concentration of X.

In the interim, a factorized induced spectrum (FS_I∆A_ of X$;\frac{{X{ }\left( {{\text{D}}0} \right)}}{{{ }\Delta A \cdot { }Fy}}$ is generated utilizing computational software through the division of the (D^0^) associated with any concentration of component X (across all wavelengths scanned)by the absorbance difference calculated between the specified wavelengths, following multiplication by the determined equality factor of component Y to get the modified absorbance difference .

Multiplying the induced numerical value of the modified absorbance of the mixture ($\Delta A \cdot { }Fy){ }$ by the recorded factorized induced spectrum of X; $\frac{{{\text{X }}\left( {{\text{D}}0} \right)}}{\Delta A \cdot Fy}$ will generate the X’s D^0^ spectrumwithin the mixture, as outlined below:

2 $$\Delta A \cdot { }Fy{ } \cdot \frac{{{\text{X }}\left( {{\text{D}}0} \right)}}{\Delta A \cdot Fy}{ } = {\text{X}} \left( {D0} \right)$$

The resolution of component Y is attained through application of the spectrum subtraction methodology, wherein the generated D^0^ spectrum of X is subtracted from the overall spectrum of the binary mixture.

### Risk analysis

The scanned D^0^ spectra of mixtures within UV region (200–400 nm) is classified into regions based on the extent of interference which varies from partially partial overlap or complete obscuration by broader spectra. Choosing the manipulated region should consider a geometrical shape of initial D^0^ and the spectral characteristics of the accompanying compound (s). Carefully choosing appropriate mathematical parameters offers advantages of enhanced selectivity, accuracy, precision and sensitivity and simplified analytical processes. Consequently, all the above mentioned factors should be taken into consideration when developing novel methods for quantifying complex binary or ternary mixtures, particularly when the target analytes of these mixtures exhibit overlapped spectra. To study the efficiency of the resolution of the applied methods at different wavelengths regions using different mixtures containing various levels of the standard of the analyzed drugs in duplicate, the obtained results of were analyzed and innovative cumulative validation score (CVS) is calculated which is total score reflect the risk degree of error based on the variability values. Strategic objectives in this CVS metric based on four pillars: bias (SE%), repeatability (RSD%), intermediate precision(RSD%) and robustness(RSD%). Bias (SE%) representing accuracy and reflects the closeness of the obtained concentration from the actual one and it is calculated using the theoretical concentrations and it was determined by the percent mean deviation from known concentration, bias % = [(Concentration found—known concentration) × 100/known concentration] while precision (RSD%) values reflects the comprehensive methodological variability, encompassing everything from sample preparation to acquisition and it were performed on the same day (repeatability)as well as within three different days(inter-mediate precision).RSD (%). Finally, the robustness measures the capacity of an analytical method to remain unaffected by small but deliberate variations in method parameters. Robustness provides some indication of the reliability of an analytical method during normal usage and it is performed as RSD% via studying the variability of ± 0.1 nm. The sum of these values for the analyzed mixtures is calculated irrespective to the signal to get CVS and the value of CVS ≤ 1 representing low risk while if the value of CVS > 1 up to 2 showing intermediate risk while > 2 confirming that high risk.

## Experimental

### Apparatus and software

Spectrophotometric measurements were carried out by a Shimadzu UV-1800 double beam spectrophotometer, employing precisely matched 1.00 cm quartz cells for the experimental procedure. Spectral scans were conducted across the wavelength range of 200.0–400.0 nm with intervals of 0.1 nm, employing a set of identical Quartz cuvettes with pathlength of 1 cm. The spectra were automatically acquired utilizing the Shimadzu UV-Probe 2.43 system software.

### Samples and solvents

CAR and HCT pure samples were provided by Global Napi Pharmaceutical Industries (Cairo, Egypt). Their purity was verified and determined to be 99.93% ± 0.62 for CAR and 99.88% ± 0.52 for HCT in accordance with their official methods^9^, respectively.

Co-Dilatrol^®^ Tablet dosage form was manufactured by Chemipharm, (Lot No. 6062171962). Each single dose comprises 25.0 mg of CAR and 12.5 mg of HCT.

Analytical—grade ethanol procured from E. Merck, located at Darmstadt, Germany, was utilized as the solvent.

### Standard solutions

Stock solutions of CAR and HCT, each with a concentration of 1.0 mg/mL, were prepared by accurately weighing each analyte and transferring it into separate 100-mL volumetric flasks, followed by dissolving in ethanol. The stability of these stock solutions was verified for a minimum of 1 month when stored at 4 °C.

Working solutions with a concentration of 100.0 µg/mL for both CAR and HCT were prepared via dilution of each corresponding stock solution with ethanol.

Various laboratory-prepared mixtures in duplicate were created by accurately transferring appropriate amounts of the targeted analytes in varying ratios from their respective working solutions into two sets of 10-mL volumetric flasks, utilizing ethanol as a solvent.

## Procedure

### Spectral properties

The D^0^ of CAR and HCT were separately scanned between 200.0 and 400.0 nm, with ethanol utilized as the blank.

### Linearity and construction of calibration graphs

Calibration standards over two concentration ranges were prepared at two different maxima for each studied drug. The recorded (D^0^) spectral absorbance values for CAR 2.0–12.0 µg/mL at 242.50 nm and 4.0–40.0 µg/mL at 285.5 nm for CAR and (1.0–11.0 µg/mL at 226.10 nm and 2.0–20.0 µg/mL at 270.5 nm for HCT were plotted in relationto their respective concentrations, and the regression equations were subsequently computed.

### Construction of factorized spectra

#### For AR and IAR of CAR

The CAR’s factorized absorbance difference spectra (FS_ΔA_) and (FS_IΔA_) were generated via software designed for spectrophotometry by dividing the (D^0^) corresponding to any CAR’s pure concentration across its linearity range by the interpoint absorbance difference (∆A) numerical value at two regions; 236.0 nm and 249.2 nm (A_236 nm_–A_249.2 nm_) and at 286.5 nm and 319.0 nm (A_286.5 nm_–A_319 nm_) were chosen in case of AR method. While in case of IAR method, the numerical values of absorbance were used at 242.5 nm and 295.6 nm, with the latter multiplied by the factor F of HCT; (∆A = A_242.5_–FA_295.6_) and at 285.5 nm and 319.0 nm, with the latter multiplied by the factor F of HCT ;(∆A = A_285.5_–FA_319.0_). Equality factor (F) of pure HCT’s spectra is calculated by averaging the ratio between the measured absorbance values at 242.5 nm (A_242.5_) and 295.6 nm (A_295.6_), as well as between 285.5 nm (A_285.5_) and 319.0 nm (A_319.0_), for various concentrations of HCT.

#### For AR of HCT

The HCT’s factorized absorbance difference spectrum (FS_ΔA_) was acquired by dividing the (D^0^) associated with any HCT’s pure concentration across its linearity range by the numerical value of the interpoint absorbance difference (∆A) at two regions; 226.1 nm and 246.2 nm (A_226.1 nm_–A_246.2 nm_) and at 270.5 nm and 288.8 nm (A_270.5 nm_–A_288.8 nm_) utilizing software designed for spectrophotometry.

### Application to laboratory prepared mixtures

Exact portions of the mentioned drugs in each combination were individually relocated from their respective working solutions into a set of 10-mL volumetric flasks. This procedure was performed to create mixtures with varying ratios of both drugs, and the flasks were then filled to the mark with ethanol. Following scanning every mixture within the range of 200.0–400.0 nm, the respective spectra of every mixture were stored in the computer system. Consequently, the manipulation steps of each suggested method were implemented utilizing the respective factorized spectrum.

#### Wavelength region I

##### AR for *CAR* coupled with SS for HCT

For each laboratory-prepared mixture, the absorbance difference (ΔA) (A_236 nm_–A_249.2 nm_)was multiplied by the respective factorized ΔA spectra (FS_ΔA_) of CAR prepared at the same wavelengths to yield the D^0^ spectra of CAR. Afterwards, the D^0^ spectra of HCT were derived by subtracting each acquired spectrum from the spectrum of the respective mixture.

##### AR for HCT coupled with SS for *CAR*

For every laboratory-prepared mixture, the absorbance difference (ΔA) between 226.1 and 246.2 nm (A_226.1 nm_–A_246.2 nm_) was multiplied by the respective factorized ΔA spectrum (FS_ΔA_) of HCT to generate the D^0^ spectra of HCT. Subsequently, the D^0^ spectra of CAR were generated by subtracting each acquired spectrum from the spectrum of the respective mixture.

#### Wavelength region II

##### IAR for *CAR* coupled with SS for HCT method

For every laboratory- prepared mixture, the absorbance difference (ΔA) between 242.5 nm and 295.6 nm, with the latter multiplied by the factor F; (A_242.5 nm_–FA_295.6 nm_) was multiplied by the factorized induced ΔA spectrum of CAR (FS_IAR_) obtaining the D^0^ spectra of CAR, subsequently, each acquired spectrum underwent subtraction from the spectrum of the respective mixture to yield the D^0^ spectra of HCT.

#### Wavelength region III

##### AR for CAR coupled with SS for HCT method

For every laboratory-prepared mixture, the absorbance difference (ΔA) between 286.5 nm and 319.0 (A_286.5 nm_–A_319.0 nm_) was multiplied by the respective factorized ΔA spectrum (FS_ΔA_) of CAR to yield the D^0^ spectra of CAR, subsequently, each acquired spectrum underwent subtraction from the spectrum of the respective mixture to derive the D^0^ spectra of HCT.

##### AR for HCT coupled with SS for CAR method

For every laboratory-prepared mixture, the absorbance difference (ΔA) between 270.5 and 288.8 nm (A_270.5 nm_–A_288.8 nm_) was multiplied by the respective factorized ΔA spectrum (FS_ΔA_) of HCT to generate the D^0^ spectra of HCT, subsequently, each acquired spectrum underwent subtraction from the spectrum of the respective mixture to yield the D^0^ spectra of CAR.

##### IAR for CAR coupled with SS for HCT method

For every laboratory-prepared mixture, the absorbance difference (ΔA) between 285.5 and 319.0 nm, with the latter multiplied by the factor F; (A_285.5 nm_–FA_319.0 nm_) was multiplied by the factorized induced ΔA spectrum of CAR (FS_IAR_) obtaining the D^0^ spectra of CAR, subsequently, each acquired spectrum underwent subtraction from the spectrum of the respective mixture to derive the D^0^ spectra of HCT.

In the context of all preceding approaches, including AR-SS, IAR-SS, the concentrations of CAR and HCT in every mixture were deduced by substituting the relevant values into the respective computed regression equations at their corresponding maxima in relation to the corresponding concentrations.

### Application to pharmaceutical preparations

Ten tablets of Co-Dilatrol^®^ were individually weighed, pulverized and meticulously mixed. A proper quantity of a single tablet was precisely relocated into a 100-mL beaker, and consequently 50.0 mL ethanol was added before being sonicated for 30 min. Afterwards, the resulting mixture was subsequently filtered via Whatman filter paper 4 into a 100-mL volumetric flask, and the volume was adjusted to the final mark utilizing ethanol, resulting in the preparation of a stock solution. Appropriate dilutions were applied to prepare two different solutions claimed to be (10.0–5.0 µg/mL) and (4.0—2.0 µg/mL) for CAR and HCT, respectively. The procedures pertaining to the application of laboratory mixtures were implemented. The concentrations of CAR and HCT were computed using the respective corresponding regression equation applicable to each method.

Standard addition technique was also implemented, involving the addition of pure standard drugs to the respective pharmaceutical preparation prior to the execution of the suggested procedures.

## Results and discussion

This study is directed towards third-generation beta-blockers, exemplified by carvedilol, known for its antihypertensive and metabolic effects. The imperative necessity of the quality control laboratories for cost-effective, uncomplicated and fast analytical methodologies suitable for the routine analysis of diverse pharmaceutical formulations has led to the widespread and distinctive adoption of the spectrophotometric approaches^32–42,45–50^.

The suggested UV methods do not necessitate the intricate treatment and procedures typically correlated with chromatographic method^61,62^ since they require minimal time and labor, and entail reduced solvent consumption with high sensitivity and selectivity.

**Figure 1:**
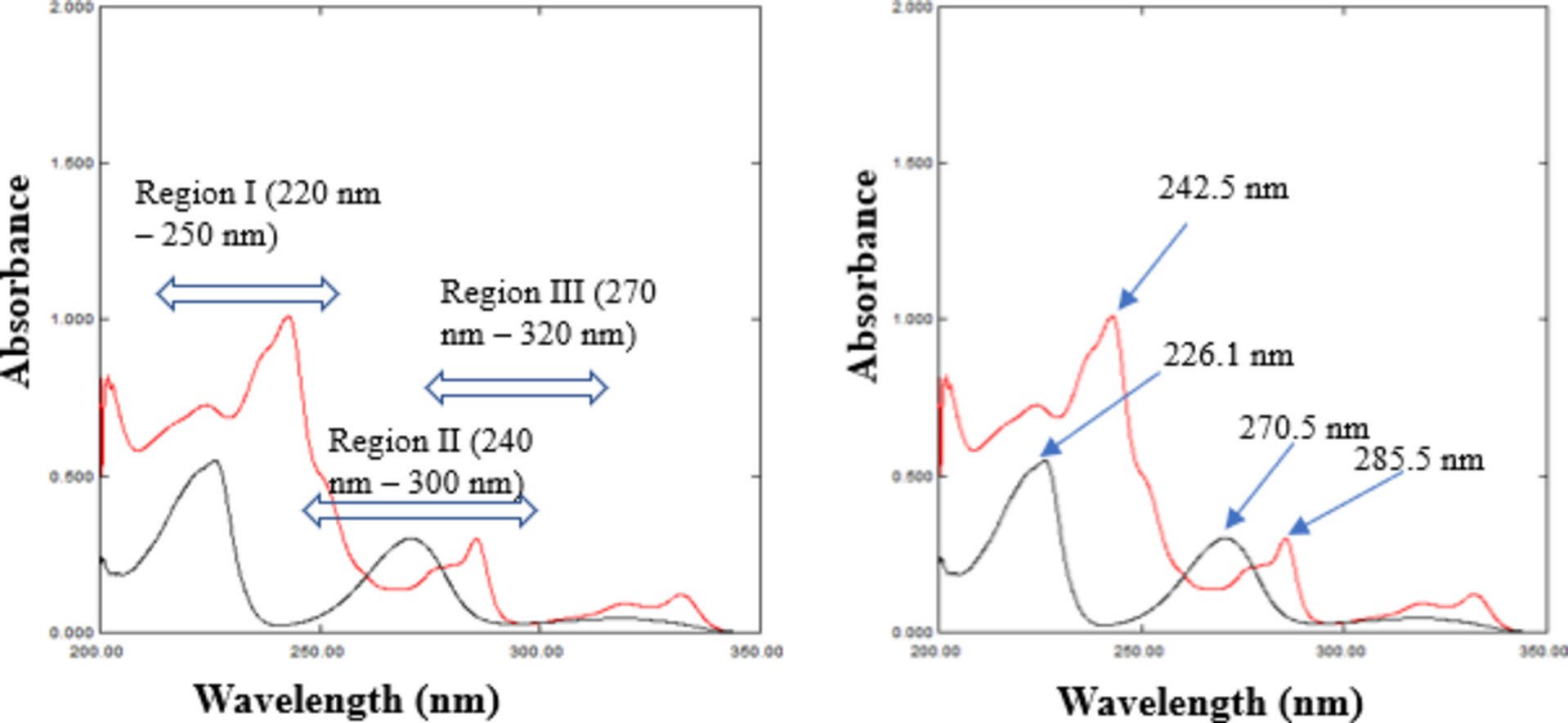
Zero order absorption spectra of 8.0 µg/mL CAR (—) and 4.0 µg/mL HCT (—) showing selected wavelength regions and their maxima.

Considering the preceding arguments and upon the examination of the zero-order absorption spectra of CAR and HCT (Fig. 1), it was noted that the spectra of the mentioned drugs overlapped. Regrettably, this spectral overlap, posed a challenge, impeding the concurrent quantification of the suggested drugs in their binary mixture. Hence, the primary objectives aimed to develop innovative spectrophotometric approaches to resolve these mixtures, emphasizing simplicity via minimizing the manipulation steps, and aiming for the ability to accurately generate the characteristic absorption spectra of the drugs under examination.

In the present study, a crucial methodology was successfully executed by employing the factorized spectra of the suggested drugs as zero order.

The D^0^ spectra of CAR and HCT; Fig. 1 showed two peaks for each of CAR and HCT with two maximum absorbance values; λ_max_ 242.5 nm and 285.5 nm for CAR and 226.1 nm and 270.5 nm for HCT. Resolution of the binary mixture of CAR and HCT is applied at three wavelength regions, leveraging the spectral features of the mentioned drugs along with their sensitivity at these regions. The three selected regions are region I (220.0–250.0 nm), region II (240.0–300.0 nm) and region III (270.0–320.0 nm).

The UV-maxima of for CAR and HCT, respectively are 242.5 nm and 226.1 nm (region I) with a linearity range of 2.0–12.0 µg/mL and 1.0–11.0 µg/mL which was suitable for the analysis of mixtures that contain low drugs' concentrations. Spectral features of the absorbance spectrum of CAR and HCT at region I (220.0–250.0 nm) showed maxima for CAR and HCT at 242.5 nm and 226.1 nm, respectively with a marked absorbance of CAR, also, it showed minima for HCT at 240.8 nm. Region II (240.0–300.0 nm) is characterized with two maxima for CAR at 242.5 nm and 285.5 nm and two minima for CAR at 265.7 nm and 297.5 nm, also, it showed one maxima and minima for HCT at 270.5 nm and 291.0 nm, respectively with a linearity range of 2.0–12.0 µg/mL for CAR and 1.0–11.0 µg/mL HCT. Furthermore, a severe overlapped spectra between the studied drugs were recognized at region II.

On the other hand, the second peaks with maximum absorbance values at 285.5 nm and 270.5 nm (region III) for CAR and HCT, respectively have a linearity range of 4.0–40.0 µg/mL for CAR and 2.0–20.0 µg/mL HCT which in turn was suitable for analyzing the mixtures that contain high drugs' concentrations.

The developed methods; AR-SS^47,60^.and IAR-SS^60^. Exploiting the factorized spectrum (FS) were employed for the concurrent quantification of CAR and HCT pharmaceutical formulations. Furthermore, a comprehensive evaluation of the merits and demerits of the novel methodologies was carried out in comparison to the well- established ones. A statistical comparison of the obtained results using the various employed factorized spectra was conducted to confirm their efficacy in determining the target analytes.

### Method optimization and development

The suggested methods were applied on the scanned laboratories prepared mixtures containing different ratios of CAR and HCT via various wavelength regions using the absorbance data at the selected wavelengths.

#### Wavelength region I

##### Absorbance resolution coupled with spectrum subtraction (AR-SS)

**Figure 2:**
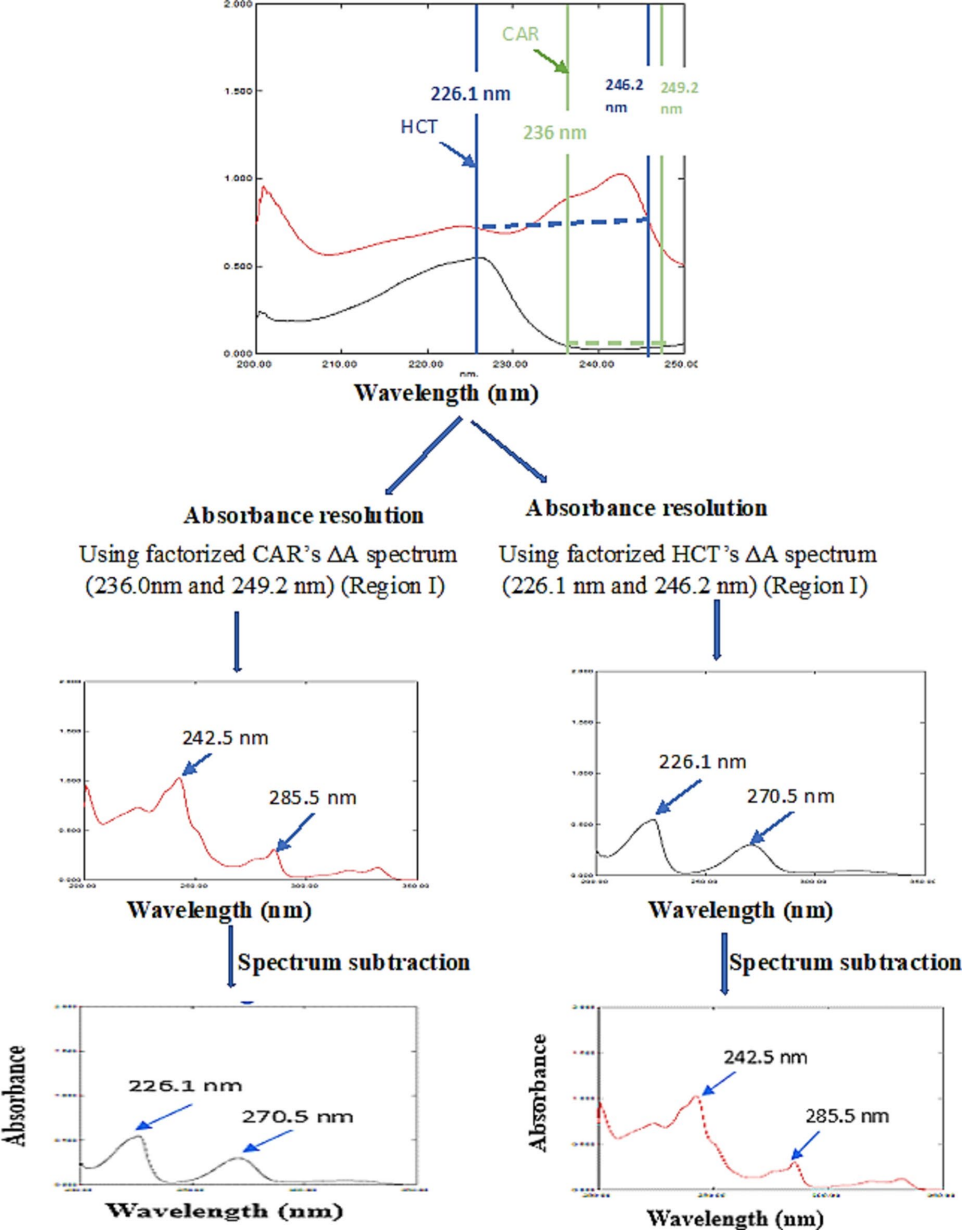
Resolution scenario of 8.0 µg/mL of CAR (—) and 4.0 µg/mL of HCT (—) in region I (220–250 nm) in their binary mixture using AR-SS .

The factorized D^0^ of CAR and HCT were obtained using D0 spectra at 236.0 nm-249.2 nm and 226.1 nm -246.2 nm for CAR and HCT, respectively, Fig. 2.

The difference in absorbance at each chosen wavelength pair was calculated in the mixture and subsequently multiplied by the aforementioned respective factorized ΔA spectrum, thus, obtaining the typical D^0^ of target drug from each mixture; Fig. 2.The acquired parent D^0^of target drug was subtracted from the gross spectrum of the respective binary mixture utilizing spectrum subtraction, yielding the parent D^0^ spectrum of co-formulated drug.

#### Wavelength region II

##### Induced absorbance resolution coupled with spectrum subtraction (IAR-SS)

**Figure 3:**
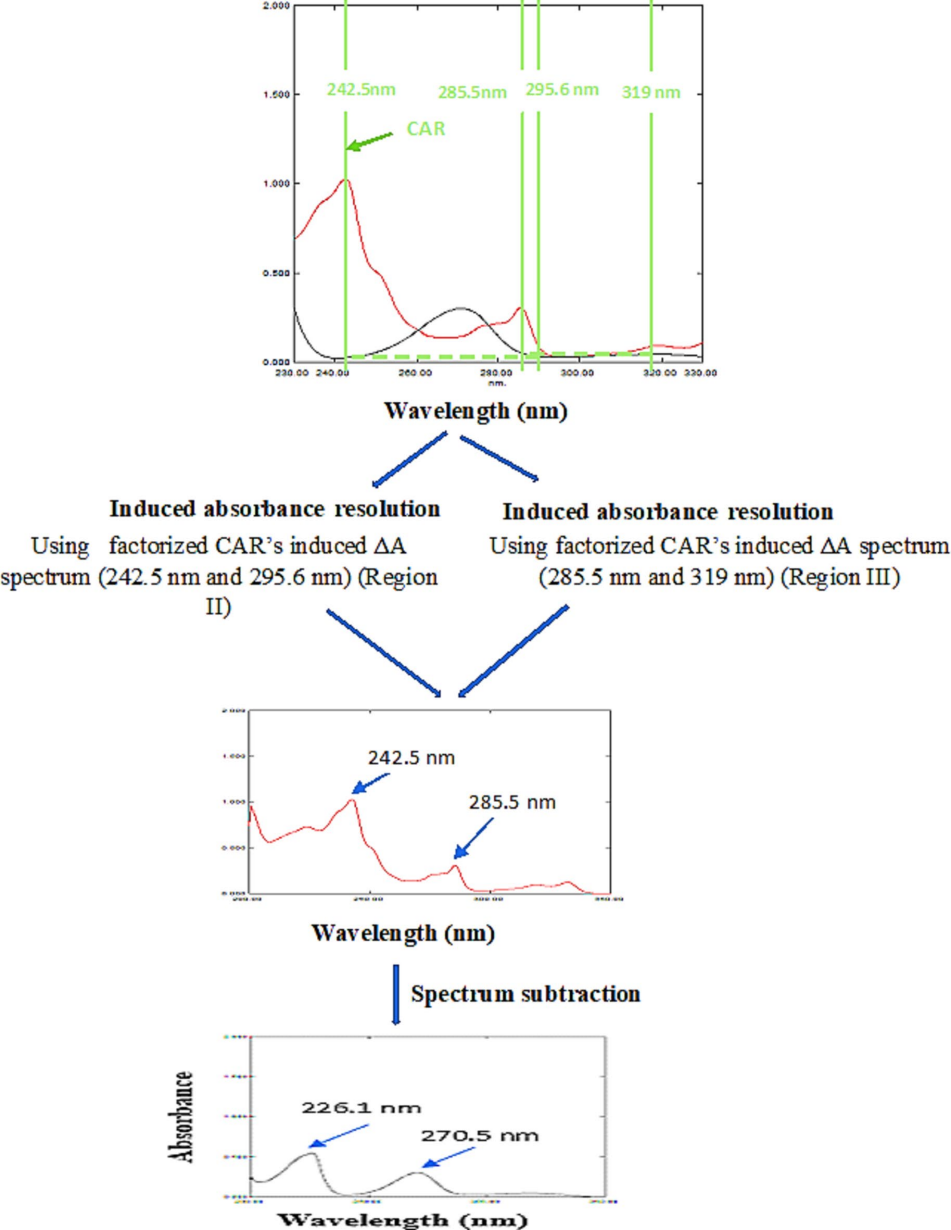
Resolution scenario of 8.0 µg/mL of CAR (—) and 4.0 µg/mL of HCT (—) in region II (240–300 nm) & region III (270–320 nm) in their binary mixture using IAR-SS.

This method was designated to improve CAR’s specificity in contrast to the AR method. Two wavelengths were selected on CAR absorption spectrum, 242.5 nm and 295.6 nm; region II. In region II, the equality factor (F) associated with pure HCT’s spectrum was computed at specific wavelength pairs, expressed as F = [A_242.5_/A_295.6_], yielding a value of 1.25.This factor ensured the equalization of absorbance for the interfering analyte (HCT) the two chosen wavelengths, whereas CAR’s absorbance exhibited a difference. At each region, the ∆A value in the D^0^ spectra of the mixtures was computed after being multiplied by the factor (F) and subsequently subjected to multiplication by the factorized induced ΔA spectrum associated with CAR, therefore, this process facilitated the reproduction of the parent D^0^ of CAR; Fig. 3.The acquired parent D^0^of CAR was drawn from the gross spectrum of the respective binary mixture employing spectrum subtraction, yielding the parent D^0^ spectrum of HCT.

#### Wavelength region III

The factorized D^0^ through the difference between two wavelengths of CAR was obtained when the absorption spectrum corresponding to concentration of 10.0 µg/mL of CAR was divided by the difference in its absorbance values 286.5–319.0 nm (A_286.5 nm_–A_319.0 nm_) for mixtures containing higher drugs' concentrations.

**Figure 4:**
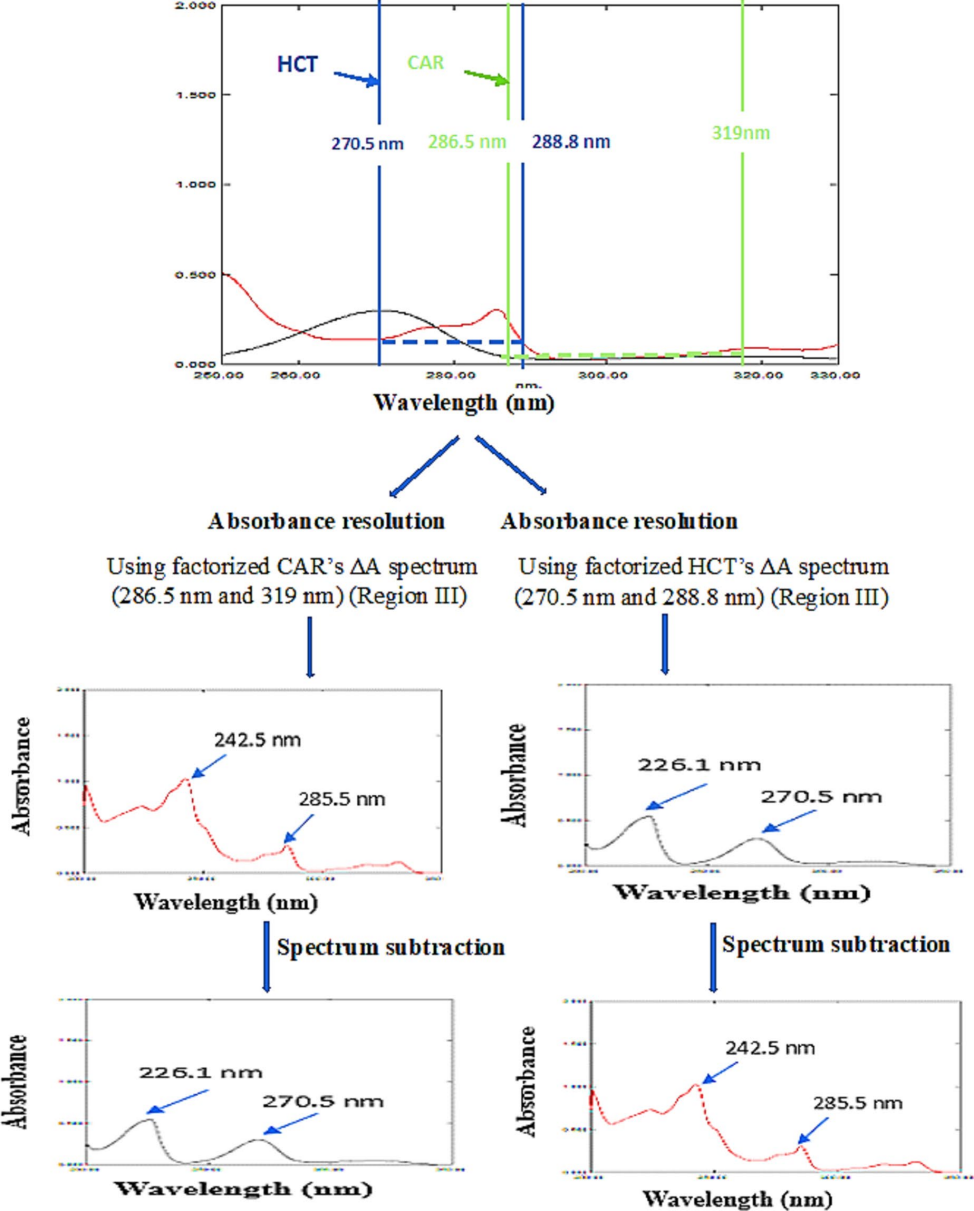
Resolution scenario of 8.0 µg/mL of CAR (—) and 4.0 µg/mL of HCT (—) in region III (270–320 nm) in their binary mixture using AR-SS.

At region III a marked absorbance difference was observed for CAR while absorbance was zero for HCT. The respective factorized ΔA CAR’s spectrum was then multiplied by the difference in absorbance at each chosen wavelength pair,thus, obtaining the typical D^0^ of CAR from each mixture; Fig. 4.The acquired parent D^0^ of CAR was drawn from the gross spectrum of the respective binary mixture employing spectrum subtraction, resulting in the parent D^0^ spectrum of HCT.

The identical steps mentioned earlier were implemented for the determination of HCT via choosing two specific wavelengths; 270.5–288.8 nm; region III for mixtures containing higher drugs' concentrations where a marked absorbance difference was observed for HCT while absorbance was zero for CAR. The difference in absorbance at each chosen wavelength pair was subsequently multiplied by the aforementioned respective factorized ΔA HCT’s spectrum, thus, obtaining the typical D^0^ of HCT from each mixture; Fig. 4. The acquired parent D^0^ of HCT was drawn from the gross spectrum of the respective binary mixture utilizing spectrum subtraction, resulting in the parent D^0^ spectrum of CAR.

##### IAR method

Two wavelengths were selected on CAR absorption spectrum at 285.5 nm and 319.0 nm. The equality factor (F) of pure HCT’s spectrum was computed at specific wavelength pairs, expressed as the (F) = [A_285.5_/A_319_], yielding a value of 1.02.This factor ensured the equalization of absorbance for the interfering analyte (HCT) the two chosen wavelengths, whereas CAR’s absorbance exhibited a difference. The ∆A value in the D^0^ spectra of the mixtures was computed after being multiplied by the factor F and subsequently subjected to multiplication by the factorized induced ΔA spectrum associated with CAR), therefore, this process facilitated the reproduction of the parent D^0^ of CAR; Fig. 3. The acquired parent D^0^ of CAR was drawn from the gross spectrum of the respective binary mixture utilizing spectrum subtraction, yielding the parent D^0^ spectrum of HCT.

For all the studied methods, CAR's and HCT's concentrations in every mixture were deduced by substituting the respective values into the respective regression equations at (242.5 nm and 226.1 nm for CAR and HCT) for low drugs' concentration ranges and (285.5 nm and 270.5 nm) for high concentration ranges; Table 1.

**Table 1:** Regression equation parameters and validation sheet obtained by applying the proposed spectrophotometric methods for the determination of CAR and HCT.

Parameter	CAR		HCT	
	AR, IAR and SS		AR and SS	
Wavelength (nm)	D^0^ (242.5 nm)	D^0^ (285.5 nm)	D^0^ (226.1 nm)	D^0^ (270.5 nm)
Calibration range (µg/mL)	2.0–12.0	4.0–40.0	1.0–11.0	2.0–20.0
Slope	0.1233	0.0365	0.1354	0.0747
SE of slope	0.000483	0.000131	0.000646	0.000272
Intercept	0.0230	0.0042	0.0145	-0.0011
SE of intercept	0.003490	0.003181	0.004464	0.003616
SD of residuals (S_y/x_)	0.00443	0.00365	0.00483	0.00379
Correlation coefficient (r)	0.9999	0.9999	0.9999	0.9999
LOD	0.12	0.33	0.12	0.17
LOQ	0.36	1.00	0.36	0.51
Accuracy (Mean ± SD)	99.99 ± 0.49	99.89 ± 0.48	99.18 ± 0.19	100.01 ± 0.80
Repeatability (RSD%)^a^	0.42	0.31	0.41	0.43
Inter-day precision (RSD%)^b^	0.39	0.48	0.72	0.56
Robustness (RSD%)^c^	0.25	0.16	0.23	0.20

^a^Relative standard deviation of three different concentrations repeated three times within the same day of CAR (2.0,6.0 and 10.0 µg/mL) and HCT (3.0,5.0 and 9.0 µg/mL) at low concentration ranges, while at high concentration ranges relative standard deviation of three concentrations of (8.0,24.0 and 32.0 µg/mL) for CAR and (6.0,14.0 and 18.0 µg/mL) for HCT.

^b^Relative standard deviation of three different concentrations repeated three times in three successive days of CAR (2.0,6.0 and 10.0 µg/mL) and HCT (3.0,5.0 and 9.0 µg/mL) at low concentration ranges, while at high concentration ranges relative standard deviation of three concentrations of (6.0,14.0 and 18.0 µg/mL) for HCT and (8.0,24.0 and 32.0 µg/mL) for CAR.

^c^Relative standard deviation of three determinations with change in scanning wavelength (± 0.1 nm).

### Risk analysis of the choice of interpoint data difference for the proposed methods

The evaluation of goodness and fitness of an analytical method is mandatory in order to ensure its intended purpose in quantification of the target drugs correctly. Method with excessive error risk will directly affect the results and provide misleading information regarding product quality.

The objective of risk analysis of different spectrophotometric strategies is to check their suitability for quantification of studied drugs with optimum accuracy and precision. Innovative cumulative validation score (CVS) is developed as the key criteria for evaluation the goodness of the strategy for analysis of mixtures containing varying ratios of target drugs. The acceptance criteria of this CVS value is cited as follows , calculated CVS ≤ 1 representing low risk where the > 1 up to 2 showing intermediate risk while > 2 confirming high risk.

In this study, the risk analysis is applied on twelve mixtures containing CAR and HCT at three wavelength region, region I (220.0–250.0 nm), region II (240.0–300.0 nm) and region III (270–320 nm) where their D^0^ spectra showing different extent of spectral contribution. Risk analysis of the choice of interpoint difference for the proposed methods using (ΔA) (226.1–246.2 nm), (236.0–249.2 nm), (242.5 nm and 295.6 nm), (270.5–288.8 nm), (286.5–319.0 nm) and (285.5–319.0 nm). The methods based on applying spectrophotometric filtration methods for analysis the proposed mixture of CAR and HCT via factorized spectrum using absorbance difference values of the component of interest to get its parent spectrum D^0^ while the D^0^ of the other drug is recovered via spectrum subtraction of gross mixture and that of the drug of interest Bias (SE%), repeatability (RSD%)_1_, intermediate precision(RSD%)_2_ and robustness(RSD%)_3_.at small variation in selected wavelengths (± 0.1 nm) during the manipulation process are calculated across various concentration levels in different mixtures and their acceptance criteria is RSD < 2% of each. The cumulative validation score (CVS) is the algebraic sum of the calculated values (SE%), (RSD%)_1_, (RSD%)_2_ and (RSD%)_3_ of each wavelength region is calculated and the results were summarized in Table 2 , Figs. S2a–c and 5.

**Table 2:** Analysis of laboratory prepared mixtures of CAR and HCT by the proposed spectrophotometric methods via window I (zero order spectrum).

No	Concentration CAR: HCT	Ratio	Window I (Zero order)											
			Wavelength region I (220–250 nm)				Wavelength region II		Wavelength region III (270–320 nm)					
							(240–300 nm)							
			CAR SS	HCT AR _226.1–246.2 nm_	CAR AR _236–249.2 nm_	HCT SS	CAR IAR _242.5–295.6 nm_	HCT	CAR SS	HCT AR _270.5–288.8 nm_	CAR AR _286.5–319 nm_	HCT SS	CAR IAR _285.5–319 nm_	HCT SS
								SS						
1	4.0:4.0	1:1	100.15	99.00	100.65	100.55	99.15	100.25	100.50	100.75	101.75	101.75	100.50	100.25
2	6.0:3.0^a^	2:1	100.00	99.90	100.83	100.67	99.00	100.67	100.45	100.63	101.67	101.33	100.67	100.67
3	4.0:8.0	1:2	99.95	100.12	101.75	101.67	99.21	100.63	99.75	100.00	101.75	100.50	100.32	100.25
4	9.0:3.0	3:1	99.86	99.00	101.44	99.33	100.00	100.67	100.07	99.33	101.56	101.33	100.25	101.33
5	2.0:6.0	1:3	99.90	100.00	100.50	100.28	99.50	100.50	NA	100.33	NA	NA	NA	NA
6	10.0:2.5	4:1	99.75	100.00	101.50	101.20	100.30	99.20	100.70	99.60	101.80	98.00	100.15	99.60
7	14.0:14.0	1:1	NA	NA	NA	NA	NA	NA	100.50	100.57	100.50	101.50	100.50	100.29
8	32.0:16.0^a^	2:1	NA	NA	NA	NA	NA	NA	100.16	99.69	100.91	100.94	99.15	100.63
9	8.0:16.0	1:2	NA	NA	101.78	NA	100.38	NA	99.15	99.33	101.25	98.56	99.25	99.06
10	30.0:10.0	3:1	NA	99.85	NA	NA	NA	NA	100.11	99.83	101.23	101.57	100.43	100.60
11	6.0:18.0	1:3	NA	NA	100.87	NA	99.23	NA	99.33	99.11	101.17	100.17	100.33	98.83
12	24.0:6.0	4:1	NA	100.66	NA	NA	NA	NA	100.15	99.50	102.00	101.17	100.25	99.67
Laboratory Prepared mixtures Mean %^b^ ± SD			99.94 ± 0.14	99.82 ± 0.56	101.17 ± 0.51	100.62 ± 0.80	99.60 ± 0.55	100.32 ± 0.57	100.08 ± 0.49	99.89 ± 0.56	101.42 ± 0.45	100.62 ± 1.25	100.16 ± 0.50	100.11 ± 0.75
Average Bias %^c^			0.07	0.18	− 1.17	− 0.62	0.40	− 0.32	− 0.08	0.11	− 1.42	− 0.62	− 0.16	− 0.11
Average RSD%^d^			0.03	0.05	0.49	0.41	0.39	0.28	0.04	0.06	1.20	0.64	0.11	0.10
Average RSD%^e^			0.04	0.07	0.61	0.81	0.42	0.41	0.07	0.08	1.40	0. 80	0.14	0.16
Robustness (± 0.1 nm ) RSD %^f^			0.19	0.31	0.98	1.12	0.85	0.61	0.27	0.38	1.49	1.41	0.11	0.18
Cumulative Validation Score (CVS)^g^			0.33	0.61	3.25	2.96	2.06	1.62	0.46	0.63	5.51	3.47	0.52	0.55

NA: Not applicable in these concentration ranges.

^a^Ratio present in dosage form. ^b^Mean of all readings for all mixtures. ^c^Average of percentage Average ^c^Bias (Relative Error (RE%) = [(Estimated concentration—Actual concentration)/Actual concentration] *100. RSD% are ^d^repeatability and ^e^intermediate precision, respectively, Average of relative standard deviation of different concentrations of each drug in the mixture. ^f^Robustness :Average of relative standard deviation of different concentrations of each drug in the mixtures using ± 0.1 nm. ^g^Cumulative Validation Score of the calculated c + d + e + f . CVS ≤ 1 representing low risk while if the value of CVS > 1 up to 2 showing intermediate risk while > 2 confirming that high risk.

**Figure 5:**
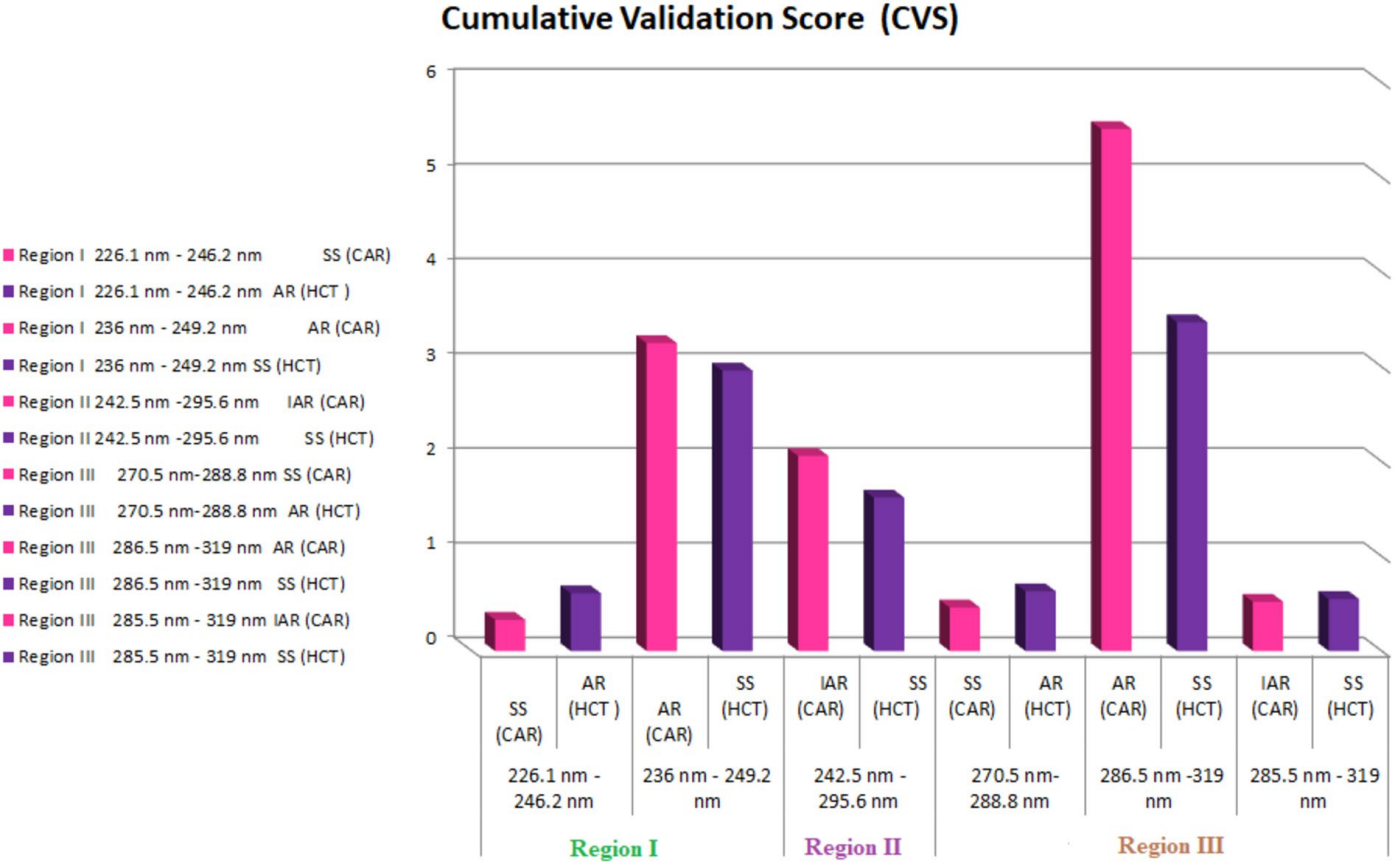
The results of Cumulative Validation Score (CVS) using AR-SS and IAR-SS in regions I, II and III.

By applying AR method via factorized HCT at the two regions, region I 220.0–250.0 nm and region III 270.0–300.0 nm then CAR is acquired through spectrum subtraction of the extracted HCT from the overall spectrum of their mixture.AR was applied between 226.1 and 246.2 nm; region I, where 226.1 nm is a maxima and 246.2 nm is a minima in HCT’s spectrum with a marked high difference in absorbance (∆A = 1.222) and the average bias % was 0.07 and 0.18 with repeatability precision 0.03% and 0.05% and intermediate precision 0.04% and 0.07% for CAR and HCT, respectively. Moreover, AR was applied at region III between 270.5 and 288.8 nm via FS of HCT, where 270.5 nm is a maxima in HCT’s spectrum and 288.8 nm is a minima in HCT’s spectrum with limited difference in the absorbance (∆A = 0.635) and the average bias (SE%) was found to be − 0.08 and 0.11 with repeatability precision 0.04% and 0.06% and intermediate precision 0.07% and 0.08% for CAR and HCT, respectively. Thus based on these results, the applying of AR via maxima and minima showing good robustness upon using ± 0.1 nm with RSD% 0.19% and 0.31% in case of region I and 0.27% and 0.38% in case of region III for CAR and HCT, respectively. Furthermore, The CVS was found to be 0.33 and 0.61 in case of region I and 0.46 and 0.63 in case of region III for CAR and HCT, respectively. The higher absorbance difference (∆A = 1.222) at region I lowering risk and enhances the bias% and precision (repeatability and intermediate precision).

For determination of CAR, AR was applied at region I between 236 and 249.2 nm, where the two selected wavelengths are shoulders in CAR’s spectrum with absorbance difference (∆A = 0.440) to get parent spectrum (D^0^) of CAR then D^0^ of HCT is obtained via spectrum subtraction and the average bias % was − 1.17 and − 0.62 with repeatability precision 0.49% and 0.41% and intermediate precision 0.61% and 0.81% for CAR and HCT, respectively. Furthermore, AR was applied at region III between 286.5 and 319 nm via FS of CAR, where 286.5 nm is a shoulder in CAR’s spectrum with ∆A = 0.236 and the average bias (SE%) was found to be − 1.42 and − 0.62 with repeatability precision 1.20% and 0.64% and intermediate precision 1.40% and 0.80% for CAR and HCT, respectively. Thus based on these results, the applying of AR via maxima and minima showing good robustness upon using ± 0.1 nm with RSD% 0.98% and 1.12% in case of region I and 1.49% and 1.41% in case of region III for CAR and HCT, respectively. Furthermore, The CVS was found to be 3.25 and 2.96 in case of region I and 5.51 and 3.47 in case of region III for CAR and HCT, respectively. The higher absorbance difference (∆A = 0.440) at region I lowering risk and enhances the bias% and precision (repeatability and intermediate precision).

Additionally, IAR was applied for determination of CAR at different regions. IAR was applied at region II between 242.5 and 295.6 nm, where 242.5 nm is a maxima and 295.6 nm is minima in CAR’s spectrum with a marked difference in absorbance (∆A = 1.218) and the average bias (SE%) was 0.40 and − 0.32 with repeatability precision 0.39% and 0.28% and intermediate precision 0.42% and 0.41% for CAR and HCT, respectively. Also, IAR was applied at region III between 285.5 and 319.0 nm, where 285.5 nm is maxima in CAR’ s spectrum with ∆A = 0.239 and the average bias % was found to be − 0.16 and − 0.11 with repeatability precision 0.11% and 0.10% and intermediate precision 0.14% and 0.16% for CAR and HCT, respectively. Thus based on these results, the applying of IAR via maxima and minima showing good robustness upon using ± 0.1 nm with RSD% 0.85% and 0.61% in case of region II and 0.11% and 0.18% in case of region III for CAR and HCT, respectively. Furthermore, The CVS was found to be 2.06 and 1.62 in case of region II and 0.52 and 0.55 in case of region III for CAR and HCT, respectively. The absorbance difference at region III using two broad peak maxima with lower absorbance difference value (∆A = 0.239) and low interference of HCT and lower equality factor (F) than that in region II equal to 1.02 (F = [A_285.5_/A_319.0_]) has a merit over that of region III with higher absorbance difference (∆A = 1.218) and higher equality factor 1.25 (F = [A_242.5_/A_295.6_]).

In analysis of mixtures containing low concentrations of CAR and HCT , both maxima for CAR (242.5 nm and 285.5 nm) and HCT (226.1 nm and 270.5 nm)could be employed for analysis, while mixtures containing high concentrations of CAR and HCT only 285.5 nm for CAR and 270.5 nm for HCT can be used .

Based on the results, the best scenarios were found to be those obtained by AR of HCT -SS of CAR at region I (220.0–250.0 nm) have CVS 0.61 and 0.33, respectively and While AR of HCT -SS of CAR at region III (270.0–320.0 nm) has CVS0.63 and 0.46, respectively while IAR of CAR –SS of HCT at region III (270.0–320.0 nm) have CVS 0.52 and 0.55, respectively. Thus, these applied methods at these regions showing low risk factor and maximum robustness as shown in Table 2 and Fig. 5.

## Advantages of the proposed methods compared to the reported spectrophotometric methods

Analysts are provided with a sophisticated tool that facilitates the extraction of valuable analytical information from spectra. Comprehension of the spectral characteristics of the studied spectra leads to proper selection of the wavelength region of analysis, enhancing the assay’s selectivity. The proposed methods AR, IAR coupling with SS methods exhibit wide applicability owing to their capability to accommodate diverse spectral features of the analyzed mixtures, involving partial or complete overlap, the presence or absence of extension, and the occurrence of isoabsorptive points and no need for divisors or derivatization, additionally, there is a minimal need for manipulation steps applied on the software designed for spectrophotometry. The principal benefit presented by the suggested methods is the factorized spectra (FS) which is the resolving spectrum dependent on its recorded response at the chosen wavelength(s) rather than concentration, as observed in normalized spectrum, so, it overcomes the random error or systematic error (instrumental error).These methods have a favor over the reported spectrophotometric methods^3,21–24^ that they have the capability to recover the D^0^ of both components within the studied mixtures, thereby representing their spectral profile and the drugs in the combination could be precisely and accurately dedicated at their λ_max_ with accuracy and precision. Two spectrophotometric resolution strategies based on using FS of one of the mentioned drugs in the mixtures were applied by getting D^0^ of CAR via mathematical resolution (AR or IAR) while the D^0^ of HCT could be obtained via SS using arithmetic manipulation (subtraction) and vice versa, thus no needs for several optimization trials for developing a complementary method for the recovering the co-formulated drug.

## Method validation

Acceptance criteria of validation parameter was reported^63–65^ to be for linearity : The correlation coefficient for six concentration levels will be ≥ 0.999, For accuracy: For the U.S. pharmaceutical industry, 100 ± 2% is typical for an assay of an active ingredient in a drug product thus % Recovery 98–102% , bias (SE%) must be < 2%, For precision repeatability (RSD%), intermediate precision (RSD%) : The assay results obtained by two operators on different days should have a statistical RSD% < 2% and robustness (RSD%) should be < 2%. The studied spectrophotometric approaches were validated following the guidelines outlined by ICH^57^, focusing on parameters such as methods' sensitivity, linearity range, accuracy, precision, LOD, LOQ and robustness with minor changes in scanning wavelength (± 0.1 nm) and these aspects were agree with the acceptance criteria as detailed in Table 1. Table 2 depicts the specificity outcomes derived from analyzing laboratory—prepared mixtures comprising varied drugs’ ratios at different wavelength regions affirming the studied methods’ specificity, indicating satisfactory outcomes throughout the calibration range.

Based on the results of risk analysis, the best scenarios with low risk obtained by AR of HCT-SS of CAR at region I (220.0–250.0 nm), AR of HCT- SS of CAR at region III (270.0–320.0 nm) and IAR of CAR –SS of HCT at region III (270.0–320.0 nm) were also successful in determining the studied drugs in Co-Dilatrol^®^ Tablets. Furthermore, assessment of the studied method’s validity was conducted by employing Standard addition technique. Therefore, these applied methods at these regions showing better recoveries upon analysis of dosage form and application of standard addition technique, as depicted in Table 3.

**Table 3:** Results obtained by applying the best scenarios for the determination of CAR and HCT in Co-Dilatrol^®^ tablets and application of standard addition technique.

	Window I (Zero order)						
	Pharmaceutical Dosage form						
	Found%^a^ ± SD						
	Wavelength region I (220–250 nm)		Wavelength region III (270–320 nm)				
	CAR SS	HCT ΔA_226.1–246.2 nm_	CAR SS	HCT ΔA_270.5–288.8 nm_	CAR ΔA_285.5–319 nm_	HCT SS	
Co-Dilatrol^®^ Tablets B. No. 070140 A; labeled to contain 25.0 mg Carvedilol and 12.5 mg Hydrochlorothiazide	100.62 ± 0.45	99.82 ± 0.31	99. 65 ± 0.27	100.78 ± 0.38	100.86 ± 0.49	100.95 ± 0.56	

Claimed (µg/mL)		Standard addition					
		Recovery %					
CAR	HCT	Wavelength region I (220–250 nm)		Wavelength region III (270–320 nm)			
		CAR (SS)	HCT (ΔA_226.1–246.2 nm_)	CAR (SS)	HCT (ΔA_270.5–288.8 nm_)	CAR (ΔA_285.5–319 nm_)	HCT (SS)
4.0	2.0	100.32	99.95	NA	NA	NA	NA
		100.75	99.87	99.75	99.71	99.67	99.05
		100.63	100.00	99.50	99.25	99.35	100.25
10.0	5.0	NA	99.75	99.80	99.60	100.13	99.60
		NA	100.12	100.12	99.40	100.25	100.17
		NA	NA	100.00	99.50	99.30	99.40
Recovery %^a^ ± SD		100.57	99.94	99.83	99.49	99.74	99.78
		± 0.22	± 0.14	± 0.24	± 0.18	± 0.44	± 0.40

In region I, added concentrations of CAR (2.0, 4.0 and 8.0 µg/mL) and HCT (1.0, 2.0 and 4.0 µg/mL). In case of region III; added concentrations of CAR (5.0, 10.0 and 20.0 µg/mL) and HCT (2.5, 5.0 and 10.0 µg/mL).

^a^Mean of three readings.

Moreover, a statistical comparison was conducted between the results acquired from the studied methods and those from the official methods^9^.The calculated t and F values were observed to be lower than their respective theoretical values, indicating that there was negligible disparity between the studied and official methods in terms of accuracy and precision, as detailed in Table S1.

A statistical analysis employing a one—way ANOVA test was conducted on the results acquired from the application of the studied approaches and those acquired by the official methodologies. In this analysis, the calculated F (F_cal_) values were observed to be lower than the tabulated F (F_tab_) values, and the P values were greater than 0.05 for both targeted analytes. These findings demonstrated that there is no substantial disparity between the studied methods and the official one, as depicted in Table S2.

## Greenness profile assessment

The GAC has recommended various approaches for creating sustainable analytical procedures instead of conventional ones. The principle of green analytical chemistry advocates for the mitigation or minimization of hazardous chemical usage in analytical approaches to bolster environmental sustainability, while maintaining method’s efficacy^49,50,66–69^. Methods’ greenness profile was ensured through various assessment tools to compare the studied methods’ greenness. Among the assessment tools employed were analytical greenness metric (AGREE), green analytical procedure index (GAPI), analytical eco-scale, and national environmental methods index (NEMI).In the studied methodologies, the analytical greenness (AGREE) metric assessment tool^49–52^ was employed, yielding a score value of 0.73 on the AGREE pictogram which was found to be greener in comparison to official methods of CAR and HCT^9^ which was found to be 0.53 and 0.57, respectively as shown in Table 4. Furthermore, a comprehensive evaluation of the method’s greenness profile was conducted using the GAPI tool^52–54^, revealing that the studied methods exhibit seven green, seven yellow and only one red zone which was also greener than official method of CAR^9^ which appeared as five green, four yellow and six red zones, while official method of HCT^9^ appeared as four green, six yellow and five red zones, as depicted in Table 4.

**Table 4:** Greenness assessment of the proposed Spectrophotometric and official methods according to AGREE, GAPI, Eco-Scale and NEMI tools.

Proposed Methods		Official methods^9^			
		CAR^a^	HCT^b^		
AGREE TOOL					
GAPI Tool					

Analytical eco-scale	Penalty points	Analytical eco-scale	Penalty points	Analytical eco-scale	Penalty points
Reagents		Reagents		Reagents	
Ethanol	4	Acetic acid	8	Tetrahydrofuran	12
		Perchloric acid	16	Methanol	12
				Phosphate buffer	0
Technique		Technique		Technique	
UV -Spectrophotometry	0	Potentiometry	0	HPLC	0
Energy Consumption	0	Energy Consumption	0	Energy Consumption	1
Occupational Hazards	0	Occupational Hazards	3	Occupational Hazards	3
Waste	3	Waste	5	Waste	3
Total penalty points	∑ 7	Total penalty points	∑ 32	Total penalty points	∑ 31
Analytical Eco-Scale total score	93	Analytical Eco-Scale total score	68	Analytical Eco-Scale total score	69
NEMI Tool					

^a^For Carvedilol: The official method using a non-aqueous potentiometric titration method using 0.1 M perchloric acid as a titrant.

^b^For Hydrochlorothiazide: The official method reported HPLC method using gradient elution of mobile phase A comprising of phosphate buffer, methanol and tetrahydrofuran (94:6:1, by volume) & mobile phase B comprising of phosphate buffer, methanol and tetrahydrofuran (50: 50:5, by volume) (pH 3.2) at flow rate 0.8 mL/min, C_18_ column (4.6 mm × 10 cm) and UV detection at 224 nm.

An additional scoring tool, which utilized the penalty point system up to 100 (representing an ideal green approach),was introduced as a semi–quantitative procedure for assessing both the ecological consequences and the eco-friendliness of the methods^55^.

The proposed methods revealed high Eco-score of 93whichwas greener than official methods of CAR and HCT^9^ which had an Eco-score of 68 and 70, respectively; Table 4.Ultimately, the evaluation of the methods’ greenness profile was conducted utilizing NEMI tool^56^, wherein all four quadrants in the suggested methods were shaded green owing to the absence of ethanol in both the PBT-list (persistent, bio accumulative, and toxic) and the TRI hazardous list, as well as its non- corrosive nature with the generation of less than 50 g of waste, therefore it was found to be more green than the official method of CAR^9^ which showed three white quadrants and only one green quadrant , while official method of HCT^9^ showed two white and two green quadrants; Table 4. The suggested spectrophotometric methods have advantages over the official methods to analyze CAR and HCT^9^. These advantages include the capability to reduce solvent usage and consume lower instrumental energy by utilizing environmentally friendly solvents, as evidenced by high scores for analytical eco-score and AGREE, as well as exhibiting full green NEMI pictogram and one red zone in GAPI pictogram.

## Conclusion

This combination has great impact with dual action as antihypertensive as well as reducing insulin resistance in diabetic patients. The studied methods are characterized with selectivity, accuracy, precision and environmental friendliness, and don’t necessitate any prior treatment for the analysis of CAR and HCT. The proposed methods allowing the recovery of D^0^ of CAR and HCT with selective determination at their respective maxima. To get low risk, AR -SS is better to be applied at interpoint difference (ΔA) between 226.1 and 246.2 nm at region I and between 270.5 and 288.8 nm at region III .While in case of IAR -SS, low risk is obtained using 285.5 nm and 319.0 nm in the manipulation steps. The studied methods were successful in the quantification of the target analytes in their mixtures as well as in their pharmaceutical preparation. The studied methods do not require extensive handling, intricate operative steps or excessive use of organic solvents associated with the costly HPLC and HPTLC approaches. Thus, the suggested methods can be regarded as alternative approaches in the quality control laboratories for the reliable analysis of drug combinations since they necessitate minimal sample preparation steps, particularly for laboratories in developing countries where the access to HPLC instrumentation is limited.

### Spectral resolution recommendation

As a final recommendation, selection of the wavelength region for manipulation and resolution scenario of low risk with respect to accuracy and precision based on the factorized spectrum via the absorbance difference either direct (AR) or induced (IAR) engaged with spectrum subtraction (SS) should be applied in the region where the D^0^ spectra of the two drugs are completely overlapped according to the following guidelines:

#### For AR-SS method

Select wavelength region in which the drug of interest show broad peak and the interfering drug show equal absorbance irrespective to intensity of the absorbance and geometrical features of the studied spectra. Starting mathematical resolution scenario via AR method in the region where the drug of interest has maxima as one of the selected wavelengths at zero difference of the interfering drug to ensure optimum robustness (± 1 nm).The other co-formulated drug is obtained by SS.

#### For IAR-SS method

Select wavelength region in which the drug of interest show maxima with highest absorbance intensity. Starting mathematical resolution scenario (IAR) in the selected region by drug of interest using two selected wavelengths one of them is the maxima and the other is minima allowing maximum absorbance difference and optimum robustness (± 1 nm) while interfering drug has the smallest equality factor at these chosen wavelengths to minimize error. The other co-formulated drug is obtained by SS.

### Supplementary Information

Supplementary Information.

## Data Availability

All data generated or analysed during this study are included in this published article.
