# Supplementary material for: Risk assessment based on spectrophotometric signals used in eco-friendly analytical scenarios for estimation of carvedilol and hydrochlorothiazide in pharmaceutical formulation
Source: Sci Rep. 2024 Aug 23;14:19657. doi: 10.1038/s41598-024-69746-0 (PMC11343851; doi:10.1038/s41598-024-69746-0)
Supplement: Supplementary file 1 — Supplementary Information. [file 41598_2024_69746_MOESM1_ESM.docx]

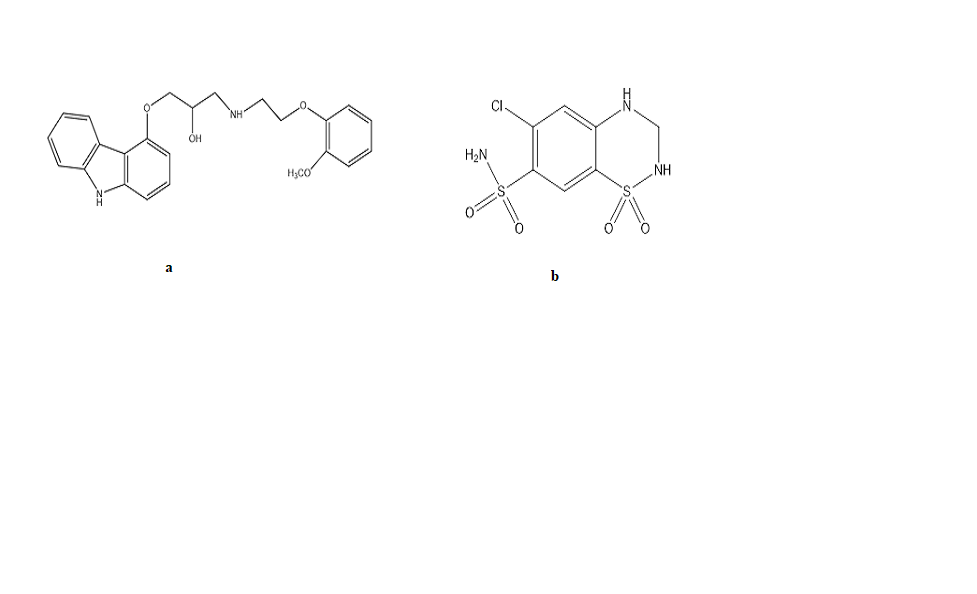
**Figure S1.** Chemical structures of a) Carvedilol (CAR) and b) Hydrochlorothiazide HCT).


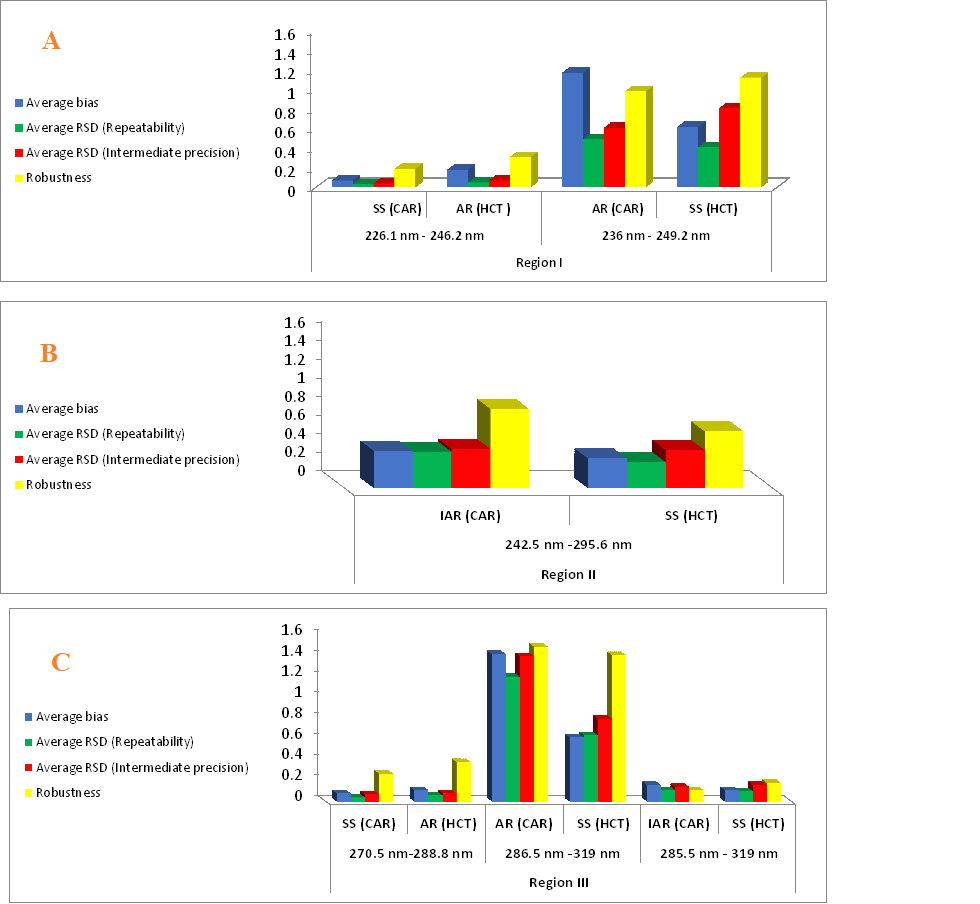


**Figure S2.** The results of average bias, average RSD (Repeatability), average RSD (Intermediate precision) and robustness obtained using AR-SS and IAR-SS in regions I, II and III.

**Table S1:** Statistical comparison between the results obtained by the proposed spectrophotometric methods and official method ^[9]^ for the determination of CAR and in pure powder forms.

| **Items** | **CAR** | | | **HCT** | | |
| --- | --- | --- | --- | --- | --- | --- |
|  | **D^0^ (242.5 nm)** | **D^0^ (285.5 nm)** | **Official  method** ^[9]^ **^a^** | **D^0^ (226.1 nm)** | **D^0^ (270.5 nm)** | **Official  method** ^[9]^ **^b^** |
| **Mean** | 99.71 | 100.07 | 99.93 | 99.78 | 99.92 | 99.88 |
| **SD** | 0.64 | 0.49 | 0.62 | 0.61 | 0.49 | 0.52 |
| **RSD** | 0.64 | 0.49 | 0.62 | 0.61 | 0.49 | 0.52 |
| **n** | 7 | 6 | 4 | 6 | 6 | 4 |
| **Variance** | 0.4096 | 0.2401 | 0.3844 | 0.3721 | 0.2401 | 0.2704 |
| **Student's-t test ^c^** | 1.423 (2.262) | 1.029 (2.306) |  | 0.772 (2.306) | 0.372 (2.306) |  |
| **F-test ^c^** | 1.066 (8.94) | 1.601 (5.41) |  | 1.376 (9.01) | 1.126 (5.41) |  |

^a^ For Carvedilol: The official method using a non-aqueous potentiometric titration method using 0.1 M perchloric acid as a titrant.

^b^ For Hydrochlorothiazide: The official method reported HPLC method using gradient elution of mobile phase A comprising of phosphate buffer, methanol and tetrahydrofuran (94:6:1, by volume) & mobile phase B comprising of phosphate buffer, methanol and tetrahydrofuran (50: 50:5, by volume) (pH 3.2) at flow rate 0.8 mL/min, C_18_ column (4.6 mm × 10 cm) and UV detection at 224 nm.

^c^ The corresponding theoretical values of t and F at P=0.05.

**Table S2**: One way ANOVA testing for the different proposed and reported methods used for the determination of the proposed mixture.

|  | **Source of Variation** | **DF** | **Sum of squares** | **Mean Square** | **F value** | **P value** |
| --- | --- | --- | --- | --- | --- | --- |
| **CAR** | **Between Groups** | 2 | 0.412 | 0.206 | 0.604 (3.740) | 0.560 |
|  | **Within Groups** | 14 | 4.775 | 0.341 |  |  |
| **HCT** | **Between Groups** | 2 | 0.062 | 0.031 | 0.104 (3.805) | 0.902 |
|  | **Within Groups** | 13 | 3.879 | 0.298 |  |  |

The values between parentheses are the theoretical F values at P ≤ 0.05.

The population means are not significantly different.
